# Prediction of Pathological Grades of Pancreatic Neuroendocrine Tumors Based on Dynamic Contrast-Enhanced Ultrasound Quantitative Analysis

**DOI:** 10.3390/diagnostics13020238

**Published:** 2023-01-09

**Authors:** Dao-Hui Yang, Juan Cheng, Xiao-Fan Tian, Qi Zhang, Ling-Yun Yu, Yi-Jie Qiu, Xiu-Yun Lu, Wen-Hui Lou, Yi Dong, Wen-Ping Wang

**Affiliations:** 1 Department of Ultrasound, Zhongshan Hospital, Fudan University (Xiamen Branch), Xiamen 361006, China; 2 Department of Ultrasound, Xinhua Hospital Affiliated to Shanghai Jiaotong University School of Medicine, Shanghai 200092, China; 3 Department of Ultrasound, Zhongshan Hospital, Fudan University, Shanghai 200092, China; 4 Department of Pancreatic Surgery, Zhongshan Hospital, Fudan University, Shanghai 200032, China

**Keywords:** dynamic contrast-enhanced ultrasound (DCE-US), quantitative parameters, pancreatic neuroendocrine tumors (pNETs), tumor histopathological grades, prediction

## Abstract

Objective: To investigate whether the dynamic contrast-enhanced ultrasound (DCE-US) analysis and quantitative parameters could be helpful for predicting histopathologic grades of pancreatic neuroendocrine tumors (pNETs). Methods: This retrospective study conducted a comprehensive review of the CEUS database between March 2017 and November 2021 in Zhongshan Hospital, Fudan University. Ultrasound examinations were performed by an ACUSON Sequioa unit equipped with a 3.5 MHz 6C−1 convex array transducer, and an ACUSON OXANA2 unit equipped with a 3.5 MHz 5C−1 convex array transducer. SonoVue^®^ (Bracco Inc., Milan, Italy) was used for all CEUS examinations. Time intensity curves (TICs) and quantitative parameters of DCE-US were created by Vuebox^®^ software (Bracco, Italy). Inclusion criteria were: patients with histopathologically proved pNETs, patients who underwent pancreatic B-mode ultrasounds (BMUS) and CEUS scans one week before surgery or biopsy and had DCE-US imaging documented for more than 2 min, patients with solid or predominantly solid lesions and patients with definite diagnosis of histopathological grades of pNETs. Based on their prognosis, patients were categorized into two groups: pNETs G1/G2 group and pNETs G3/pNECs group. Results: A total of 42 patients who underwent surgery (n = 38) or biopsy (n = 4) and had histopathologically confirmed pNETs were included. According to the WHO 2019 criteria, all pNETs were classified into grade 1 (G1, n = 10), grade 2 (G2, n = 21), or grade 3 (G3)/pancreatic neuroendocrine carcinomas (pNECs) (n = 11), based on the Ki−67 proliferation index and the mitotic activity. The majority of the TICs (27/31) of pNETs G1/G2 were above or equal to those of pancreatic parenchyma in the arterial phase, but most (7/11) pNETs G3/pNECs had TICs below those of pancreatic parenchyma from arterial phase to late phase (*p* < 0.05). Among all the CEUS quantitative parameters of DCE-US, values of relative rise time (rPE), relative mean transit time (rmTT) and relative area under the curve (rAUC) were significantly higher in pNETs G1/G2 group than those in pNETs G3/pNECs group (*p* < 0.05). Taking an rPE below 1.09 as the optimal cut-off value, the sensitivity, specificity and accuracy for prediction of pNETs G3/pNECs from G1/G2 were 90.91% [58.70% to 99.80%], 67.64% [48.61% to 83.32%] and 85.78% [74.14% to 97.42%], respectively. Taking rAUC below 0.855 as the optimal cut-off value, the sensitivity, specificity and accuracy for prediction of pNETs G3/pNECs from G1/G2 were 90.91% [66.26% to 99.53%], 83.87% [67.37% to 92.91%] and 94.72% [88.30% to 100.00%], respectively. Conclusions: Dynamic contrast-enhanced ultrasound analysis might be helpful for predicting the pathological grades of pNETs. Among all quantitative parameters, rPE, rmTT and rAUC are potentially useful parameters for predicting G3/pNECs with aggressive behavior.

## 1. Introduction

The incidence of pancreatic neuroendocrine tumors (pNETs), which are heterogeneous tumors that arise from the pancreas′s diffuse neuroendocrine cell system, is less than 1 per 100,000 per year [1,2]. According to the 2019 World Health Organization (WHO) recommendations, pNETs are classified as grade 1 (G1), grade 2 (G2), grade 3 (G3) and pancreatic neuroendocrine carcinomas (pNECs) based on the Ki-67 proliferation index and the mitotic activity [3]. The pathological grade of pNETs is one of the most important factors in the management of pNETs and is highly correlated with the survival rate [4]. According to the current European Neuroendocrine Tumor Society (ENETS) criteria, radical resection is mandatory for all pNETs larger than 2 cm and for functioning tumors [5,6,7]. Surgeries such as central pancreatectomy and enucleation are proposed for pNETs G1 patients, which have excellent prognosis and a long survival time after surgery [5,8]. Due to the high risk of recurrence, adjuvant chemotherapy is advised for pNETs G3 and pNECs following resection [6,9]. Different systemic therapies should be recommended for unresectable pNETs, based on pathological grading, for improved disease control and symptom relief. Arterial chemoembolization (TACE), radiofrequency ablation (RFA), peptide receptor-targeted radiotherapy (PRRT) and somatostatin analogs are all advised in unresectable pNETs G1/2; however, in unresectable pNETs G3/pNECs, only cisplatinum/etoposide is advised [10,11,12]. Tumor grades are usually assessed preoperatively by fine-needle aspiration. Disagreements of pathological grade between a biopsy and resected specimen are possible due to the heterogeneity of pNETs and the limited amount of specimen obtained by biopsy [13,14,15]. Therefore, other features, including ultrasound imaging, are required for predicting the pNETs’ pathological grade preoperatively.

Various imaging methods have previously been used to predict the histopathological grades of pNETs. The computerized tomography (CT) scan demonstrated that the enhancement ratio in the arterial phase was negatively correlated with tumor grades (*p* < 0.01). The sensitivity and specificity for pNETs G3 identification were 94% and 92%, respectively, when taking cut-off values of 1.06 [16]. The sensitivity, specificity and accuracy of CT for predicting the pathological grades of pNETs was around 73–91%, 85–100% and 65–95%, respectively [16,17,18,19]. Magnetic resonance imaging (MRI) features, including apparent diffusion coefficient (ADC) values and true diffusion (D) coefficient values, were significantly lower as grades increased (ADC: 2.13 ± 0.70, 1.78 ± 0.72 and 0.86 ± 0.22 10 mm^2^/s, and D: 1.92 ± 0.70, 1.75 ± 0.74 and 0.82 ± 0.19 10 mm^2^/s, G1, G2 and G3, all *p* < 0.001) [20]. Sensitivity and specificity of MRI for predicting pNET grades were 71–95% and 87–91%, respectively [20,21,22]. However, these are all very initial clinical pilot studies, with limitations including the subjective interpretation of imaging findings and lack of dynamic perfusion characteristics in lesions. Meanwhile, the efficacy of CT and MRI is constrained by drawbacks such as cost, probable radiation exposure and severe contrast agent allergy. 

Contrast-enhanced ultrasound (CEUS) was the only imaging method that allowed for real-time evaluation of the microcirculation perfusion by the strictly intravascular blood pool agent, which was different to CT or MRI contrast agent [23,24,25]. It has been shown in recent decades to be an effective imaging modality for improving the diagnosis performance of pancreatic tumors, including preoperative differential diagnosis of pNETs and treatment effect monitoring in pancreatic cancer [26,27,28]. Dynamic contrast-enhanced ultrasound (DCE-US) and quantitative parameters also enable one to objectively analyze the microvascularization of non-cystic lesions in terms of characterization and detection. It was used for predicting microvascular invasion and assessing therapeutic efficacy in hepatocellular carcinoma (HCC) patients. The peak intensity of lesions (25.4 ± 9.6) before transcatheter arterial chemoembolization (TACE) was significantly higher than that (15.3 ± 5.1) three days after TACE (*p* < 0.05), which corresponded to the changes in circulating angiogenic factors in HCC patients [29,30]. It has been proven that pNETs with different histopathological grades have differences in tumor microvascular perfusion [31]. According to current EFSUMB recommendations, DCE-US is useful for quantifying tumor enhancement objectively, to characterize focal lesions and evaluate the therapeutic response [32]. Compared to CEUS, DCE-US showed advantages such as objectively evaluating the enhancement between normal and abnormal parenchyma, offering the potential to better understand the angiogenesis of HCC, renal cell carcinoma and breast cancer [29,33,34]. It might also have the potential to predict histopathological grades of pNETs based on the real-time dynamic evaluation of the microcirculation of pNETs.

The purpose of this study was to investigate whether DCE-US analysis and quantitative parameters could be helpful for predicting tumor histopathologic grades of pNETs.

## 2. Patients and Methods

### 2.1. Study Design

This retrospective study was approved by our institutional review board (ID: B2021-144). Informed consent was waived. The procedure followed was in accordance with the Declaration of Helsinki. 

### 2.2. Patients

We conducted a comprehensive review of the CEUS database between March 2017 and November 2021 in Zhongshan Hospital, Fudan University. 

Inclusion criteria were: patients with histopathologically proved pNETs, patients who underwent pancreatic B-mode ultrasounds (BMUS) and CEUS scans one week before surgery or biopsy and had DCE-US imaging documented for more than 2 min, patients with solid or predominantly solid lesions and patients with definite diagnosis of histopathological grades of pNETs. Exclusion criteria were: pancreatic tumors that could not be visualized clearly on transabdominal BMUS, patients with a lack of final histopathological grade diagnosis of the pancreatic lesion and patients who underwent radiotherapy, chemotherapy or other treatments before surgery/biopsy. 

Patients in our study were categorized into 2 groups based on the National Comprehensive Cancer Network (NCCN) guidelines depending on their aggressive behavior and prognosis [35]: pNETs G1/G2 group and pNETs G3/pNECs group. In our study, the cut-off values of cancer biomarkers in serum were determined by the People’s Republic of China health standard (http://www.nhc.gov.cn/wjw/wsbzxx/wsbz_9.shtml) accessed on 1 June 2019.

### 2.3. CEUS Study Protocol

All patients fasted at least 8 h before ultrasound examinations. Ultrasound examinations were performed by an ACUSON Sequoia unit (Siemens Medical Solutions, Germany) equipped with a 3.5 MHz 6C-1 convex array transducer, or an ACUSON OXANA2 unit (Siemens Medical Solutions, Germany) equipped with a 3.5 MHz 5C-1 convex array transducer. The pancreas was first evaluated using BMUS to detect and locate the lesion. Then the target pancreatic lesion was placed in the center of screen. A bolus of 1.5 mL Sonovue (Bracco Inc., Milan, Italy) was administered via the antecubital vein and followed by a 5–10 mL saline flush. All the ultrasound examinations were performed by one of two experienced radiologists with 5 years and 10 years of clinic experience in pancreatic CEUS. DICOM cine loops were continuously stored for 120 s after the injection of contrast agent for further analysis.

### 2.4. Imaging Data Analysis 

The following BMUS features were observed and documented: (1) the size and location of the pancreatic lesion; (2) echogenicity of the lesion (hypoechoic, isoechoic or hyperechoic), compared with the surrounding pancreas parenchyma; (3) presence of the pancreatic main duct dilatation (defined as a duct diameter of ≥3 mm); (4) internal color doppler flow imaging (CDFI) signals (present or absent); (5) tumor texture (solid, solid–cystic or complex cystic); (6) tumor margin (well-defined or ill-defined). 

The following CEUS features were observed and documented from arterial phase (0–30 s) to venous phase (30–120 s) to late phase (>120 s) according to current EFSUMB guidelines [24]: (1) enhancement degree of pancreatic lesion (hyperenhancement, isoenhancement or hypoenhancement), compared with the surrounding pancreas parenchyma; (2) enhancement pattern (homogeneous or heterogeneous) of pancreatic lesion; (3) internal nonenhanced area of solid lesions (present or absent).

### 2.5. Quantitative Analysis 

Quantitative analysis was performed by Vuebox^®^ software (Bracco, Italy), an external offline perfusion software. The 2 min CEUS DICOM loops were transferred to an offline computer for further analysis. A region of interest (ROI) was placed manually in a solid part of the pancreatic lesion and another ROI was placed in the surrounding pancreatic parenchyma. The motion compensation function was applied to reduce the breath motion artifact. Time intensity curves (TICs) were generated and compared between 2 groups. The quantitative analysis was performed by a skilled radiologist with 2 years of experience who was blind to pathological grades.

After curve fitting, various CEUS quantitative parameters were obtained, including the rise time (RT), peak intensity (PE), time to peak (TP), mean transit time (mTT) and area under the curve (AUC) (Table 1). The results were considered reliable when the quality of fitting exceeded 75%.

The ratio of quantitative parameters between pancreatic lesions and surrounding parenchyma were also calculated, including relative rise time (rRT), relative peak intensity (rPE), relative time to peak (rTTP), relative mean transit time (rmTT) and relative area under the curve (rAUC). S, seconds; a.u, arbitrary unit.

### 2.6. Histological Analysis

The formalin-fixed specimens were processed into paraffin sections, and the sections were stained with hematoxylin and eosin for conventional histology analysis. Immunohistochemistry was performed and according to the WHO 2019 criteria [3], pNETs G1 (low-grade) had a mitotic rate from 0 to 1 per 10 high power fields (HPF) or a Ki-67 proliferation index from 0% to 2%; pNETs G2 (intermediate-grade) had a mitotic rate from 2 to 20 per 10 HPF or a Ki-67 proliferation index from 3% to 20%; pNETs G3/pNECs (high-grade) had a mitotic rate greater than 20 per 10 HPF or a Ki-67 proliferation index greater than 20%.

### 2.7. Statistical Analyisis

Univariate analyses were performed to determine the predictors of pathological grades of pNETs through the chi-squared or the Fisher exact test for categorical variables and *t* student, and the Mann–Whitney test for continuous variables. Mean and SD were used if the outcome was approximately normal, whereas median, with the 25th and 75th percentiles, was used if the outcome was a skewed distribution. Qualitative data including clinical features, B-mode ultrasound and CEUS features were assessed by using chi-squared and the Fisher exact test for expected frequencies less than five. Cut-off values were determined by a receiver operating characteristic (ROC) analysis for diagnoses based on TIC. *p*-values < 0.05 were considered significant. Clinical utility index (CUI) provided the degree to which a diagnostic test was useful in clinical practice, including a positive (CUI+) and negative (CUI−) clinical utility index [36]. Analyses were performed with IBM SPSS version 22.0 (Armonk, NY, USA; IBM Corp), and MedCalc version 19.0.4 (MedCalc Software, Ltd., Ostend, Belgium).

## 3. Results

### 3.1. Patient Demographics

A total of 42 patients were finally enrolled. There were 10 lesions classified as pNETs G1, 21 as pNETs G2 and 11 as G3/pNECs. Patient demographics are summarized in Table 2. No significant difference was found in age and gender between pNETs G1/G2 and pNETs G3/pNECs (*p* > 0.05).

### 3.2. B-Mode Ultrasound

The majority of both pNETs G1/G2 and pNETs G3/pNECs were hypoechoic solid lesions on BMUS. There was no significant difference between the two groups in size, margin, pancreatic duct dilation or internal CDFI signals (*p* < 0.05). B-mode ultrasound imaging features are summarized in Table 3.

### 3.3. Contrast-Enhanced Ultrasound

After injection of the contrast agent, although the majority of pNETs G3/pNECs (9/11) exhibited heterogeneous enhancement, no statistically significant difference was observed when compared to pNETs G1/G2 (*p* = 0.075). There was no significant difference between pNETs G1/G2 and pNETs G3/pNECs in enhanced degree in the arterial phase (*p* = 0.069). Nonenhanced necrosis area was detected in four pNETs G1/G2 and three pNETs G3/pNECs lesions (*p* = 0.353). CEUS imaging features are summarized in Table 4.

### 3.4. Time Intensity Curves 

**Figure 1:**
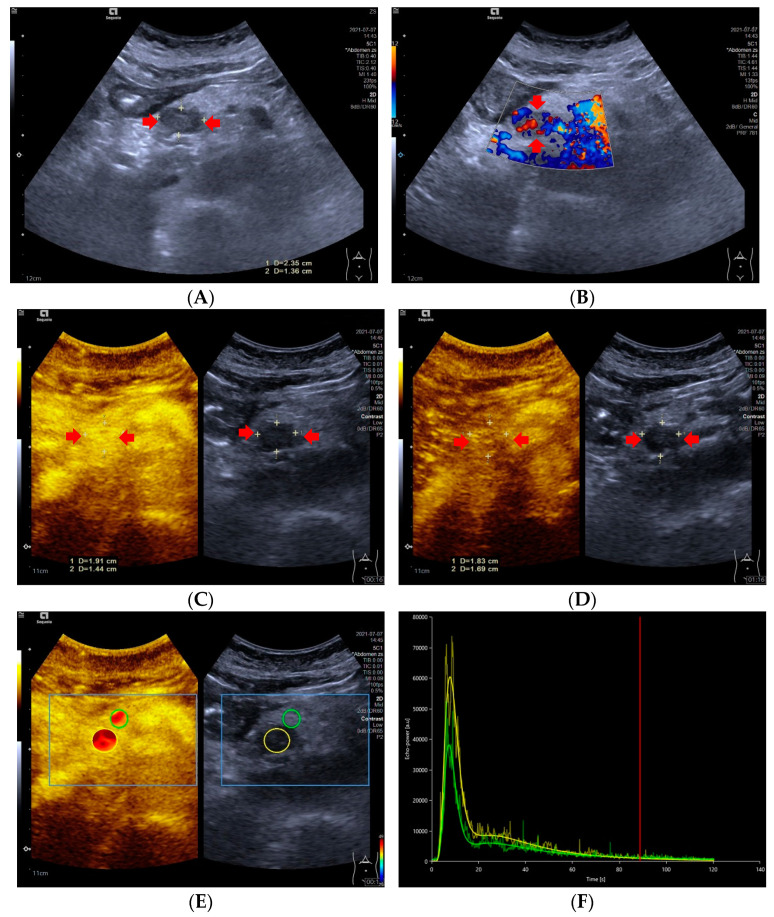
A 59-year-old female with a pNETs G1 lesion (Ki 67 proliferation index of 2%). The pNETs lesion located in pancreatic head was hypoechoic (**A**) with abundant internal color doppler flow imaging signal (**B**). After injecting the contrast agent, pNETs lesion showed hyperenhancement in the arterial phase (**C**) and isoenhancement in the venous phase (**D**). The pNET’s microvascularization was assessed using VueBox^®^, an external perfusion software. Regions of interest of the pNETs lesion (yellow circle) and surrounding pancreatic parenchyma (green circle) were placed manually (**E**). The time intensity curve revealed that in the arterial phase, the yellow curve (pNETs lesion) was higher than the green curve (pancreatic parenchyma), and in the venous phase and late phase, the two curves almost completely overlapped (**F**).

**Figure 2:**
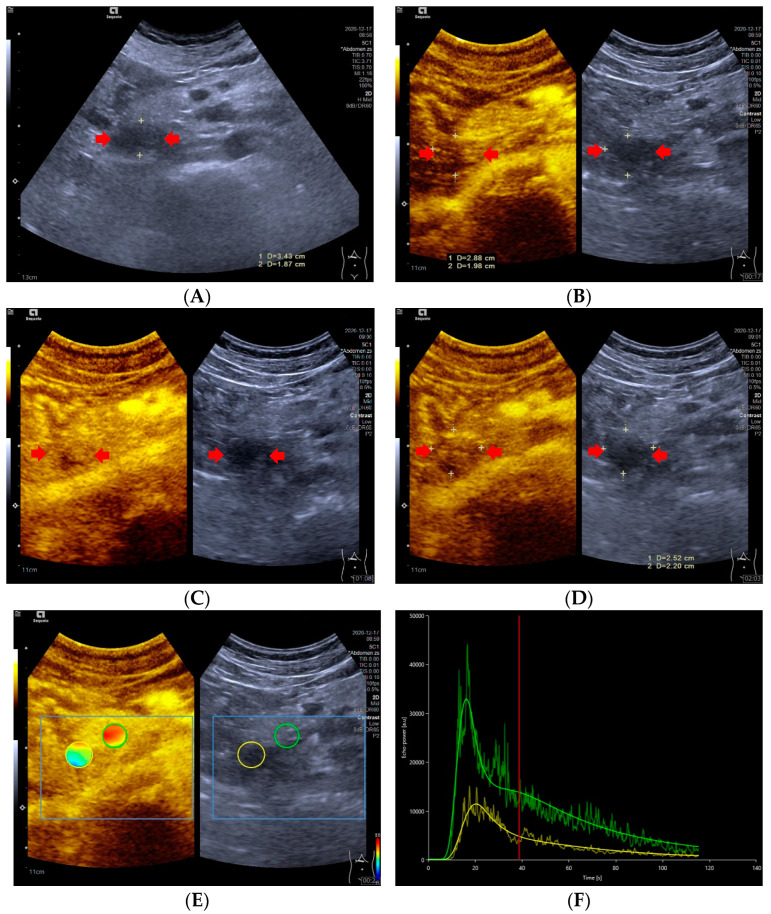
A 47-year-old male with a pNECs lesion (Ki 67 proliferation index, 80%). The pancreatic head-located pNECs lesion was hypoechoic with well-defined margins (**A**). The pNECs lesion showed heterogeneous hypoenhancement throughout arterial phase to late phase after the contrast agent injection (**B**–**D**). The regions of interest of pNECs lesion (yellow circle) and surrounding pancreatic parenchyma (green circle) were placed manually for evaluation of the pNEC’s microvascularization utilizing external perfusion software VueBox^®^ (**E**). The time intensity curve revealed that the yellow curve (pNECs lesion) was below the green curve (pancreatic parenchyma) from arterial phase to late phase (**F**).

Significant differences in TIC shape (*p* < 0.05) were observed between pNETs G1/G2 and pNETs G3/pNECs. In the group of pNETs G1/G2, 22 (22/31, 70.79%) TICs were persistently above or equal to TICs of pancreatic parenchyma from the arterial phase to the late phase. Five TICs of pNETs G1/G2 were above those of pancreatic parenchyma in the arterial phase, but below the TICs of pancreatic parenchyma in the venous phase or late phase due to the rapid wash-out of contrast agent [Figure 1]. On the contrary, seven cases (7/11, 63.64%) of pNETs G3/pNECs had TICs below those of pancreatic parenchyma consistently after injecting contrast agent [Figure 2].

### 3.5. Dynamic Contrast-Enhanced Ultrasound and Quantitative Parameters

**Figure 3:**
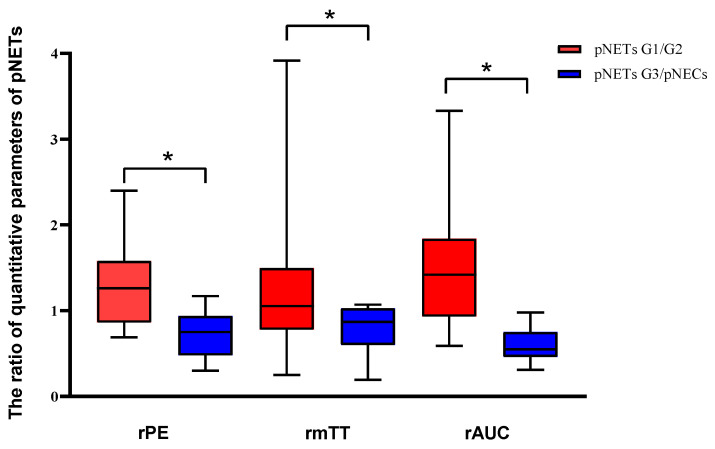
Analysis of the differences in dynamic contrast-enhanced ultrasound and quantitative parameters between pNETs G1/G2 and pNETs G3/pNECs. The relative peak intensity values, relative mean transit time values and relative area under the curve values were significantly higher in pNETs G1/G2 than those in pNETs G3/pNECs (*p* < 0.05). Asterisk (*) indicates significant difference (*p* < 0.05).

After curve fittings, significant differences were observed between the two groups in rPE, rmTT and rAUC (*p* < 0.05). The values of rPE, rmTT and rAUC in pNETs G1/G2 were significantly higher than those in pNETs G3/pNECs [Figure 3]. No statistical difference was found between pNETs G1/G2 and pNETs G3/pNECs in rRT and rTTP (*p* > 0.05). The features of DCE-US and quantitative parameters are summarized in Table 5.

### 3.6. Optimal Cut-Off Value for Prediction of Pancreatic Neuroendocrine Tumors’ Pathological Grades

**Figure 4:**
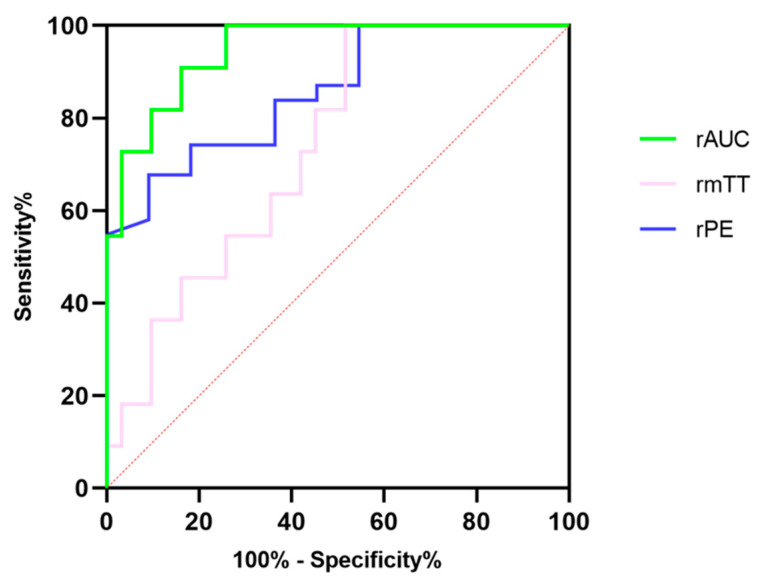
The diagnostic efficacy of relative peak intensity, relative mean transit time and relative area under the curve for differentiating pNETs G1/G2 from pNETs G3/pNECs.

Further ROC analysis was performed to evaluate the diagnostic efficacy of DCE-US quantitative parameters, for which results are summarized in Table 6. The rAUC showed the best diagnostic performance among three quantitative parameters with the highest specificity, at 83.87% and the highest accuracy at 94.72%, although the sensitivity in the rAUC was consistent with that in rPE [Figure 4]. The CUI values, including CUI+ and CUI- of the rAUC, were also higher than those of rPE and rmTT. 

## 4. Discussion

Our study elucidated that the DCE-US was a helpful and noninvasive imaging method to preoperatively predict the pathological grades of pNETs with satisfactory diagnostic performance. The quantitative parameters of rPE, rmTT and rAUC were found to be a strong reflection of the pathological grades of pNETs which should be highly considered as an adjunct factor for predicting pathological grades of pNETs preoperatively.

Dynamic contrast-enhanced ultrasound analysis based on transabdominal scans was an easier method to assess the microcirculation of pancreatic lesions and it was beneficial in monitoring the treatment response in locally advanced pancreatic tumors, according to our own prior research [27,28]. Overall, the DCE-US analysis and quantitative parameters showed a satisfactory performance for predicting the pathological grades of pNETs in our study; the pNETs G1/G2 had significantly higher rPE, rmTT, and rAUC values than pNETs G3/pNECs, indicating that the internal contrast agent in pNETs G3/pNECs lesions washed out more quickly than that of pNETs G1/G2 lesions. According to previous studies, the AUC values and PE values were parameters related to the hemodynamics of ROI on DCE-US [32,37].As well as being correlated to the degree of vascularization and density of the vascular network, fast flow and low tissue vascular resistance were available in high AUC values [38]. Compared with pNETs G3/pNECs, the higher rPE and rAUC in pNETs G1/G2 were the results of the relatively higher microvessel density (MVD) but lower fibrotic components, as already reported in several studies [39,40,41,42,43]. Hence, the rAUC and rPE were promising factors for predicting pathological grades of pNETs by evaluating microvessel perfusion quantitatively. In addition, the mTT was also a promising parameter correlated to both hemodynamics and vascular morphology [37]. Anant et al. reported that increased mean values of mTT after neoadjuvant chemotherapy were helpful to assess the response in breast cancer by the change in tumor vascularity, with an accuracy of 86.7% [44]. However, the rmTT had a lower diagnostic performance, a wider confidence interval and lower bounds of the confidence interval compared with rPE and rAUC in our study. As for our study, the metric of CUI values including CUI+ and CUI− were used for evaluating the diagnosis accuracy of predicting pNETs G3/pNECs and pNETs G1/G2. The CUI+ value of 0.61 and CUI− value of 0.82 in rAUC supported fair utility and good utility in clinical practice of predicting pNETs G3/pNECs.

Takada et al. reported that the quantitative analysis showed a high diagnostic accuracy of more than 95% for grade diagnosis of pNETs [45]. The major difference between previous and present studies was the measurement of quantitative parameters. Previous studies by Takada et al. measured only the quantitative parameters of lesions and the ROI of lesions was not placed at the same depth. As for our study, both the quantitative parameters of lesions and surrounding parenchyma were measured and the ratio of lesions with surrounding parenchyma were calculated. The ratio of quantitative parameters could lessen the effect of varying ROI depth for results because placed ROI depth was a factor correlated to the results of quantitative analysis [32,46]. To overcome this issue, the ratio of quantitative parameters was applied to predict pNETs’ pathological grades and has been approved with satisfactory diagnostic performance.

Time intensity curves were a useful method to objectively exhibit the difference between lesions and parenchyma in the enhancement degree [46,47]. In our study, the majority of TICs in pNETs G1/G2 were higher than or equivalent to TICs of pancreatic parenchyma in the arterial phase, which was consistent with the degree of enhancement shown on CEUS. On the contrary, pNETs G3/pNECs commonly displayed TICs below those of the pancreatic parenchyma and hypoenhancement on CEUS. Two pNECs were hypoenhanced in the arterial phase on CEUS, but their TICs were almost equal to those of pancreatic parenchyma. This mismatch between TICs and CEUS in the same two pNECs may be caused by the subjective analysis on CEUS. The TICs were a promising technique to observe the perfusion properties of pNETs lesions by overcoming subjective evaluation of the enhancement features.

Previous studies exhibited that the tumor size of pNETs was an important risk factor for the prediction of pathological grades and prognosis [17]. The pNETs less than 2 cm were recommended to be managed conservatively due to benign behavior [48,49]. However, in our study one of the pNETs G2 and three pNECs were less than 2 cm and had metastases. In addition, no significant difference was observed between pNETs G1/G2 and pNETs G3/pNECs in the tumor size, despite the median tumor size of the latter being larger than that of the former. Reviews of the National Cancer Data Base databases also clearly showed that only 41.1% of pNETs less than 2 cm were pNETs G1 and that nearly 30% of them were pNETs G2 and pNETs G3/pNECs with lymph nodes involved [50,51]. Therefore, making decisions about treatment strategy for pNETs on the basis of tumor size only was quite contradictory.

In our study, most pNETs showed hyper- or isoenhancement in the arterial phase on CEUS, which was consistent with previous studies, owing to their abundant vascularization [24,52]. Previous studies found that the degree of homogeneity was correlated with pathological grades of pNETs, and heterogenous enhancement in tumors showed a lower Ki-67 proliferation index compared with homogeneous enhancement cases [31,42]. However, there was no significant difference between pNETs G1/G2 and pNETs G3/pNECs concerning enhancement patterns in our study. Huang et al. reported that statistical difference was only observed between pNETs G1 and pNETs G2, but not between pNETs G1 or G2 and pNETs G3 [52]. Several studies revealed that the heterogeneous pattern resulted from cystic degeneration and had a lack of association with tumor grades [53,54]. Therefore, predicting tumor grades of pNETs by enhancement pattern on CEUS still remains controversial because of conflicting evidence in the literature.

## 5. Limitations

Our study had several limitations. First, our study was a single-center retrospective study with a relatively small sample size, especially for pNETs G3/pNECs, for which only 11 cases could be analyzed. A further multicenter study with a larger sample size should be performed. Second, four pNECs were determined via fine-needle aspiration biopsy or core needle biopsy. However, overestimation was significantly less frequent with respect to underestimation (3.5% vs. 14.7%, *p* < 0.05) [14]. Therefore, the pNETs G3/pNECs had less possibility of being overestimated. Third, the quantitative analysis was performed by only a radiologist blind to pathological grades, and we did not evaluate the reproducibility of the DCE-US in our study. Reproducibility of DCE-US clinically was important as it would affect the clinical application of this technique. Nevertheless, several published data suggest that reproducibility of DCE-US is acceptable for the clinical practice [46,55]. Fourth, pNETs G3 and pNECs were classified as one category in our study, whereas they were split into two categories in the WHO 2017 grading system. However, both pNETs G3 and pNECs were categorized as pNETs G3 in the WHO grading system before 2017. Studies show that the prognosis of pNETs G3 and pNECs is significantly worse than that of pNETs G1 and G2 [56]. In addition, microvessel density related to blood flow was notably correlated with pathological grades of pNETs, which was lower in pNETs G3/pNECs compared with pNETs G1/G2 [57]. Quantitative parameters and TIC were strongly relevant to hemodynamics [37]. Therefore, we thought it was feasible and reasonable for DCE-US to differentiate pNETs G1/G2 from pNETs G3/pNECs.

## 6. Conclusions

The DCE-US analysis and quantitative parameters have the potential value to predict the pathological grades of pNETs noninvasively. Several CEUS quantitative parameters, including the relative PE and the relative AUC, may be valuable parameters for predicting the pathological grades of pNETs.

## Figures and Tables

**Table 1:** Explanations of contrast-enhanced ultrasound quantitative parameters calculated from the TIC.

Parameter	Abbreviation	Definition
Rise time	RT (s)	Time from 10% to 90% of maximum intensity
Peak intensity	PE (a.u)	Max value of the curve
Time to peak	TTP (s)	Time from baseline intensity to maximum intensity
Mean transit time	mTT (s)	Time from the rising of the intensity up to decrease to 50% of maximum
Area under the curve (a.u)	AUC (a.u)	Calculated integral for the time intensity curve

**Table 2:** Patient demographics for pNETs.

Characteristic	pNETs G1/G2 (n = 31, %)	pNETs G3/pNECs (n = 11, %)	*p* Value
Age (years)	52.38 ± 9.37	51.45 ± 14.07	0.806
Gender			
Male	11 (35.48)	6 (54.5)	0.268
Female	20 (65.51)	5 (45.5)	
Elevated CA19-9 (>34 U/mL)	0/31 (0.0)	3/11 (27.3)	0.014
Elevated NSE (>16.3 ng/mL)	1/31 (3.2)	7/11 (63.64)	<0.001

pNETs, pancreatic neuroendocrine tumors; CA19-9, carbohydrate antigen 19-9; NSE, neuron specific enolase.

**Table 3:** B-mode ultrasound imaging features of pNETs.

Characteristic	pNETs G1/G2 (n = 31, %)	pNETs G3/pNECs (n = 11, %)	*p* Value
Tumor size (mm)	25.00 [14.00, 36.00]	32.00 [17.00, 60.00]	0.125 *
Lesion location			
Head	11 (35.5)	6 (54.5)	0.666
Body	12 (38.7)	3 (27.3)	
Tail	8 (25.8)	2 (18.2)	
Echogenicity			
Isoechoic	4 (12.9)	0 (0.0)	0.558
Hypoechoic	27 (87.1)	11 (100.0)	
Texture			
Solid	27 (87.1)	8 (72.7)	0.353
Solid-cystic	4 (12.9)	3 (27.3)	
Margin			
Well-defined	17 (54.8)	3 (27.3)	0.166
Ill-defined	14 (45.2)	8 (72.7)	
Main Pancreatic duct dilation (> 3 mm)			
Present	3 (9.7)	3 (27.3)	0.314
Absent	28 (90.3)	8 (72.7)	
CDFI Signals of tumor			
Present	11 (35.5)	5 (45.5)	0.559
Absent	20 (64.5)	6 (54.5)	

* Mann-Whitney test; BMUS, B-mode ultrasound; CDFI, color doppler flow imaging.

**Table 4:** Contrast-enhanced ultrasound imaging features of pNETs.

Characteristic	pNETs G1/G2 (n = 31, %)	pNETs G3/pNECs (n = 11, %)	*p* Value
Enhancement pattern			
Homogeneous	17 (54.8)	2 (18.2)	0.075
Heterogenous	14 (45.2)	9 (81.8)	
Enhanced degree in arterial phase			
Hyperenhancement	18 (58.1)	3 (27.3)	0.069
Isoenhancement	9 (29.0)	3 (27.3)	
Hypoenhancement	4 (12.9)	5 (45.5)	
Nonenhancement area			
Present	4 (12.9)	3 (27.3)	0.353
Absent	27 (87.1)	8 (72.7)	

**Table 5:** Comparison of DCE-US quantitative parameters between two groups.

CEUS Quantitative Parameters	pNETs G1/G2 (n = 31)	pNETs G3/pNECs (n = 11)	*p* Value
rPE	1.26 [0.86 to 1.58]	0.75 [0.48 to 0.94]	0.002
rRT	1.05 [0.78 to 1.21]	0.93 [0.70 to 1.04]	0.065
rTTP	1.00 [0.92 to 1.17]	0.91 [0.78 to 1.04]	0.083
rmTT	1.07 [0.84 to 1.50]	0.87 [0.60 to 1.03]	0.029
rAUC	1.41 [0.93 to 1.84]	0.55 [0.46 to 0.75]	<0.001

rPE, relative peak intensity; rRT, relative rise time; rTTP, relative time to peak; rmTT, relative mean transit time; rAUC, relative area under the curve (rAUC).

**Table 6:** Diagnostic performance of quantitative parameters for prediction of pancreatic neuroendocrine tumors’ pathological grades.

Variables	Cut-Off Value	Sensitivity (%) (95% CI)	Specificity (%) (95% CI)	Accuracy (%) (95% CI)	CUI+	CUI−
rPE	1.09	90.91 [58.70 to 99.80]	67.64 [48.61 to 83.32]	85.78 [74.14 to 97.42]	0.45	0.65
rmTT	1.011	72.73 [43.33 to 90.25]	58.06 [40.77 to 75.58]	73.61 [58.14 to 89.08]	0.28	0.50
rAUC	0.855	90.91 [66.26 to 99.53]	83.87 [67.37 to 92.91]	94.72 [88.30 to 100]	0.61	0.82

CUI, clinical utility index; CI, confidence interval.

## Data Availability

Data available on request due to ethical restrictions. The data presented in this study are available on request from the corresponding author. The data are not publicly available due to ethical restrictions.

## References

[1] Cives M., Strosberg J.R. (2018). Gastroenteropancreatic Neuroendocrine Tumors. CA Cancer J. Clin..

[2] Michael M., Thursfield V., Te Marvelde L., Kong G., Hicks R.J. (2021). Incidence, prevalence, and survival trends for neuroendocrine neoplasms in Victoria, Australia, from 1982 to 2019: Based on site, grade, and region. Asia Pac. J. Clin. Oncol..

[3] Nagtegaal I.D., Odze R.D., Klimstra D., Paradis V., Rugge M., Schirmacher P., Washington K.M., Carneiro F., Cree I.A., The WHO Classification of Tumours Editorial Board (2020). The 2019 WHO classification of tumours of the digestive system. Histopathology.

[4] Strosberg J.R., Cheema A., Weber J., Han G., Coppola D., Kvols L.K. (2011). Prognostic validity of a novel American Joint Committee on Cancer Staging Classification for pancreatic neuroendocrine tumors. J. Clin. Oncol.

[5] Falconi M., Eriksson B., Kaltsas G., Bartsch D.K., Capdevila J., Caplin M., Kos-Kudla B., Kwekkeboom D., Rindi G., Klöppel G. (2016). ENETS Consensus Guidelines Update for the Management of Patients with Functional Pancreatic Neuroendocrine Tumors and Non-Functional Pancreatic Neuroendocrine Tumors. Neuroendocrinology.

[6] Scott A.T., Howe J.R. (2019). Evaluation and Management of Neuroendocrine Tumors of the Pancreas. Surg. Clin. N. Am..

[7] Partelli S., Bartsch D.K., Capdevila J., Chen J., Knigge U., Niederle B., van Dijkum E.J.N., Pape U.-F., Pascher A., Ramage J. (2017). ENETS Consensus Guidelines for Standard of Care in Neuroendocrine Tumours: Surgery for Small Intestinal and Pancreatic Neuroendocrine Tumours. Neuroendocrinology.

[8] Hain E., Sindayigaya R., Fawaz J., Gharios J., Bouteloup G., Soyer P., Bertherat J., Prat F., Terris B., Coriat R. (2019). Surgical management of pancreatic neuroendocrine tumors: An introduction. Expert Rev. Anticancer Ther..

[9] Kunz P.L., Reidy-Lagunes D., Anthony L.B., Bertino E.M., Brendtro K., Chan J.A., Chen H., Jensen R.T., Kim M.K., Klimstra D.S. (2013). Consensus guidelines for the management and treatment of neuroendocrine tumors. Pancreas.

[10] Oberg K., Knigge U., Kwekkeboom D., Perren A., ESMO Guidelines Working Group (2012). Neuroendocrine gastro-entero-pancreatic tumors: ESMO Clinical Practice Guidelines for diagnosis, treatment and follow-up. Ann. Oncol..

[11] Oberg K.E., Reubi J., Kwekkeboom D.J., Krenning E.P. (2010). Role of somatostatins in gastroenteropancreatic neuroendocrine tumor development and therapy. Gastroenterology.

[12] Kulke M.H., Shah M.H., Benson A.B., Bergsland E., Berlin J.D., Blaszkowsky L.S., Emerson L., Engstrom P.F., Fanta P., Giordano T. (2015). Neuroendocrine tumors, version 1.2015. J. Natl. Compr. Canc. Netw..

[13] Rebours V., Cordova J., Couvelard A., Fabre M., Palazzo L., Vullierme M.P., Hentic O., Sauvanet A., Aubert A., Bedossa P. (2015). Can pancreatic neuroendocrine tumour biopsy accurately determine pathological characteristics?. Dig. Liver Dis..

[14] Tacelli M., Bina N., Crinò S.F., Facciorusso A., Celsa C., Vanni A.S., Fantin A., Antonini F., Falconi M., Monica F. (2022). Reliability of grading preoperative pancreatic neuroendocrine tumors on EUS specimens: A systematic review with meta-analysis of aggregate and individual data. Gastrointest. Endosc..

[15] Facciorusso A., Mohan B.P., Crinò S.F., Ofosu A., Ramai D., Lisotti A., Chandan S., Fusaroli P. (2021). Contrast-enhanced harmonic endoscopic ultrasound-guided fine-needle aspiration versus standard fine-needle aspiration in pancreatic masses: A meta-analysis. Expert Rev. Gastroenterol. Hepatol..

[16] Guo C., Zhuge X., Wang Z., Wang Q., Sun K., Feng Z., Chen X. (2019). Textural analysis on contrast-enhanced CT in pancreatic neuroendocrine neoplasms: Association with WHO grade. Abdom. Radiol..

[17] Liang W., Yang P., Huang R., Xu L., Wang J., Liu W., Zhang L., Wan D., Huang Q., Lu Y. (2019). A Combined Nomogram Model to Preoperatively Predict Histologic Grade in Pancreatic Neuroendocrine Tumors. Clin. Cancer Res..

[18] Gu D., Hu Y., Ding H., Wei J., Chen K., Liu H., Zeng M., Tian J. (2019). CT radiomics may predict the grade of pancreatic neuroendocrine tumors: A multicenter study. Eur. Radiol..

[19] Canellas R., Burk K.S., Parakh A., Sahani D.V. (2018). Prediction of Pancreatic Neuroendocrine Tumor Grade Based on CT Features and Texture Analysis. Am. J. Roentgenol..

[20] Lotfalizadeh E., Ronot M., Wagner M., Cros J., Couvelard A., Vullierme M.-P., Allaham W., Hentic O., Ruzniewski P., Vilgrain V. (2017). Prediction of pancreatic neuroendocrine tumour grade with MR imaging features: Added value of diffusion-weighted imaging. Eur. Radiol..

[21] Kulali F., Semiz-Oysu A., Demir M., Segmen-Yilmaz M., Bukte Y. (2018). Role of diffusion-weighted MR imaging in predicting the grade of nonfunctional pancreatic neuroendocrine tumors. Diagn. Interv. Imaging.

[22] De Robertis R., Cingarlini S., Martini P.T., Ortolani S., Butturini G., Landoni L., Regi P., Girelli R., Capelli P., Gobbo S. (2017). Pancreatic neuroendocrine neoplasms: Magnetic resonance imaging features according to grade and stage. World J. Gastroenterol..

[23] Sofuni A., Tsuchiya T., Itoi T. (2020). Ultrasound diagnosis of pancreatic solid tumors. J. Med. Ultrason..

[24] Sidhu P.S., Cantisani V., Dietrich C.F., Gilja O.H., Saftoiu A., Bartels E., Bertolotto M., Calliada F., Clevert D.-A., Cosgrove D. (2018). The EFSUMB Guidelines and Recommendations for the Clinical Practice of Contrast-Enhanced Ultrasound (CEUS) in Non-Hepatic Applications: Update 2017 (Long Version). Ultraschall Med..

[25] Li X.-Z., Song J., Sun Z.-X., Yang Y.-Y., Wang H. (2018). Diagnostic performance of contrast-enhanced ultrasound for pancreatic neoplasms: A systematic review and meta-analysis. Dig. Liver Dis..

[26] Yang D., Wang D., Qiu Y., Tian X., Zuo D., Dong Y., Lou W., Wang W. (2022). Incidental nonfunctioning pancreatic neuroendocrine tumors: Contrast enhanced ultrasound features in diagnosis1. Clin. Hemorheol. Microcirc..

[27] Zuo D., Feng Y., Zhang Q., Qiu Y.-J., Tian X.-F., Shi S.-N., Dong Y., Liu T.-S., Wang W.-P. (2021). The value of dynamic contrast enhanced ultrasound (DCE-US) in monitoring treatment effect of high-intensity focused ultrasound (HIFU) in locally advanced pancreatic cancer (LAPC). Clin. Hemorheol. Microcirc..

[28] Zhang Q., Wu L., Yang D., Qiu Y., Yu L., Dong Y., Wang W.-P. (2020). Clinical application of dynamic contrast enhanced ultrasound in monitoring the treatment response of chemoradiotherapy of pancreatic ductal adenocarcinoma. Clin. Hemorheol. Microcirc..

[29] Tian H., Wang Q. (2016). Quantitative analysis of microcirculation blood perfusion in patients with hepatocellular carcinoma before and after transcatheter arterial chemoembolisation using contrast-enhanced ultrasound. Eur. J. Cancer.

[30] Dong Y., Qiu Y., Yang D., Yu L., Zuo D., Zhang Q., Tian X., Wang W.-P., Jung E.M. (2021). Potential application of dynamic contrast enhanced ultrasound in predicting microvascular invasion of hepatocellular carcinoma. Clin. Hemorheol. Microcirc..

[31] Khanna L., Prasad S.R., Sunnapwar A., Kondapaneni S., Dasyam A., Tammisetti V.S., Salman U., Nazarullah A., Katabathina V.S. (2020). Pancreatic Neuroendocrine Neoplasms: 2020 Update on Pathologic and Imaging Findings and Classification. Radiographics.

[32] Dietrich C.F., Averkiou M.A., Correas J.-M., Lassau N., Leen E., Piscaglia F. (2012). An EFSUMB introduction into Dynamic Contrast-Enhanced Ultrasound (DCE-US) for quantification of tumour perfusion. Ultraschall Med..

[33] Wildner D., Pfeifer L., Goertz R.S., Bernatik T., Sturm J., Neurath M.F., Strobel D. (2014). Dynamic contrast-enhanced ultrasound (DCE-US) for the characterization of hepatocellular carcinoma and cholangiocellular carcinoma. Ultraschall Med..

[34] Lassau N., Coiffier B., Kind M., Vilgrain V., Lacroix J., Cuinet M., Taieb S., Aziza R., Sarran A., Labbe-Devilliers C. (2016). Selection of an early biomarker for vascular normalization using dynamic contrast-enhanced ultrasonography to predict outcomes of metastatic patients treated with bevacizumab. Ann. Oncol..

[35] Shah M.H., Goldner W.S., Benson A.B., Bergsland E., Blaszkowsky L.S., Brock P., Chan J., Das S., Dickson P.V., Fanta P. (2021). Neuroendocrine and Adrenal Tumors, Version 2.2021, NCCN Clinical Practice Guidelines in Oncology. J. Natl. Compr. Canc. Netw..

[36] Bolboaca S.D. (2019). Medical Diagnostic Tests: A Review of Test Anatomy, Phases, and Statistical Treatment of Data. Comput. Math. Methods Med..

[37] Hudson J.M., Williams R., Tremblay-Darveau C., Sheeran P.S., Milot L., Bjarnason G.A., Burns P.N. (2015). Dynamic contrast enhanced ultrasound for therapy monitoring. Eur J. Radiol..

[38] Kersting S., Konopke R., Kersting F., Volk A., Distler M., Bergert H., Saeger H., Grützmann R., Bunk A. (2009). Quantitative perfusion analysis of transabdominal contrast-enhanced ultrasonography of pancreatic masses and carcinomas. Gastroenterology.

[39] Horiguchi S., Kato H., Shiraha H., Tsutsumi K., Yamamoto N., Matsumoto K., Tomoda T., Uchida D., Akimoto Y., Mizukawa S. (2017). Dynamic computed tomography is useful for prediction of pathological grade in pancreatic neuroendocrine neoplasm. J. Gastroenterol. Hepatol..

[40] Chen M.-H., Yeh Y.-C., Shyr Y.-M., Jan Y.-H., Chao Y., Li C.-P., Wang S.-E., Tzeng C.-H., Chang P.M.-H., Liu C.-Y. (2013). Expression of gremlin 1 correlates with increased angiogenesis and progression-free survival in patients with pancreatic neuroendocrine tumors. J. Gastroenterol..

[41] Kuiper P., Hawinkels L.J., De Jonge-Muller E.S., Biemond I., Lamers C.B., Verspaget H.W. (2011). Angiogenic markers endoglin and vascular endothelial growth factor in gastroenteropancreatic neuroendocrine tumors. World J. Gastroenterol..

[42] Palazzo M., Napoléon B., Gincul R., Pioche M., Pujol B., Lefort C., Fumex F., Hautefeuille V., Fabre M., Cros J. (2018). Contrast harmonic EUS for the prediction of pancreatic neuroendocrine tumor aggressiveness (with videos). Gastrointest. Endosc..

[43] Battistella A., Partelli S., Andreasi V., Marinoni I., Palumbo D., Tacelli M., Lena M.S., Muffatti F., Mushtaq J., Capurso G. (2022). Preoperative assessment of microvessel density in nonfunctioning pancreatic neuroendocrine tumors (NF-PanNETs). Surgery.

[44] Sharma A., Grover S.B., Mani C., Ahluwalia C. (2021). Contrast enhanced ultrasound quantitative parameters for assessing neoadjuvant chemotherapy response in patients with locally advanced breast cancer. Br. J. Radiol..

[45] Takada S., Kato H., Saragai Y., Muro S., Uchida D., Tomoda T., Matsumoto K., Horiguchi S., Tanaka N., Okada H. (2019). Contrast-enhanced harmonic endoscopic ultrasound using time-intensity curve analysis predicts pathological grade of pancreatic neuroendocrine neoplasm. J. Med. Ultrason..

[46] Lu Q., Huang B.-J., Xue L.-Y., Fan P.-L., Wang W.-P. (2015). Differentiation of Renal Tumor Histotypes: Usefulness of Quantitative Analysis of Contrast-Enhanced Ultrasound. Am. J. Roentgenol..

[47] Yamamoto N., Kato H., Tomoda T., Matsumoto K., Sakakihara I., Noma Y., Horiguchi S., Harada R., Tsutsumi K., Hori K. (2016). Contrast-enhanced harmonic endoscopic ultrasonography with time-intensity curve analysis for intraductal papillary mucinous neoplasms of the pancreas. Endoscopy.

[48] Sadot E., Reidy-Lagunes D.L., Tang L.H., Do R.K.G., Gonen M., D’Angelica M.I., DeMatteo R.P., Kingham T.P., Koerkamp B.G., Untch B.R. (2016). Observation versus Resection for Small Asymptomatic Pancreatic Neuroendocrine Tumors: A Matched Case-Control Study. Ann. Surg. Oncol..

[49] Bettini R., Partelli S., Boninsegna L., Capelli P., Crippa S., Pederzoli P., Scarpa A., Falconi M. (2011). Tumor size correlates with malignancy in nonfunctioning pancreatic endocrine tumor. Surgery.

[50] Kuo E.J., Salem R.R. (2013). Population-level analysis of pancreatic neuroendocrine tumors 2 cm or less in size. Ann. Surg. Oncol..

[51] Jutric Z., Grendar J., Hoen H.M., Cho S.W., Cassera M.A., Newell P.H., Hammill C.W., Hansen P.D., Wolf R.F. (2017). Regional Metastatic Behavior of Nonfunctional Pancreatic Neuroendocrine Tumors: Impact of Lymph Node Positivity on Survival. Pancreas.

[52] Huang J., Chen J., Xu M., Zheng Y., Lin M., Huang G., Xie X., Xie X. (2021). Contrast-Enhanced Ultrasonography Findings Correlate with Pathologic Grades of Pancreatic Neuroendocrine Tumors. Ultrasound Med. Biol..

[53] Kawamoto S., Johnson P.T., Shi C., Singhi A.D., Hruban R.H., Wolfgang C.L., Edil B.H., Fishman E.K. (2013). Pancreatic neuroendocrine tumor with cystlike changes: Evaluation with MDCT. Am. J. Roentgenol..

[54] Poultsides G.A., Huang L., Chen Y., Visser B.C., Pai R.K., Jeffrey R.B., Park W.G., Chen A.M., Kunz P.L., Fisher G.A. (2012). Pancreatic neuroendocrine tumors: Radiographic calcifications correlate with grade and metastasis. Ann. Surg. Oncol..

[55] Nylund K., Sævik F., Leh S., Pfeffer F., Hausken T., Gilja O.H. (2019). Interobserver Analysis of CEUS-Derived Perfusion in Fibrotic and Inflammatory Crohn’s Disease. Ultraschall Med..

[56] Wang J., Liu J., He C., Sun T., Yan Y., Che G., Li X., Sun H., Ma H. (2021). Trends in Incidence and Survival of Patients with Pancreatic Neuroendocrine Neoplasm, 1987–2016. J. Oncol..

[57] D’Assignies G., Couvelard A., Bahrami S., Vullierme M.-P., Hammel P., Hentic O., Sauvanet A., Bedossa P., Ruszniewski P., Vilgrain V. (2009). Pancreatic endocrine tumors: Tumor blood flow assessed with perfusion CT reflects angiogenesis and correlates with prognostic factors. Radiology.

